# Genital and Oral HPV Geno-Prevalence Measured through Urine and Saliva Samples in Young Adults in Italy

**DOI:** 10.3390/vaccines12020205

**Published:** 2024-02-16

**Authors:** Francesco Napolitano, Silvia Angelillo, Aida Bianco, Gabriella Di Giuseppe, Valeria Di Onofrio, Francesca Licata, Giorgio Liguori, Carmelo Giuseppe Angelo Nobile, Maria Pavia, Concetta Paola Pelullo, Federica Zito Marino, Italo Francesco Angelillo

**Affiliations:** 1 Department of Experimental Medicine, University of Campania “Luigi Vanvitelli”, 80138 Naples, Italy; 2 Department of Health Sciences, University “Magna Græcia” of Catanzaro, 88100 Catanzaro, Italy; 3 Department of Medical and Surgical Sciences, University “Magna Græcia” of Catanzaro, 88100 Catanzaro, Italy; 4 Department of Sciences and Technologies, University of Naples “Parthenope”, 80143 Naples, Italy; 5 Department of Medical, Movement and Wellbeing Sciences, University of Naples “Parthenope”, 80133 Naples, Italy; 6 Department of Pharmacy, Health and Nutritional Sciences, University of Calabria, 87036 Cosenza, Italy; 7 Department of Mental and Physical Health and Preventive Medicine, University of Campania “Luigi Vanvitelli”, 80138 Naples, Italy

**Keywords:** geno-prevalence, HPV vaccination, saliva, urine, young adults

## Abstract

Background: The aims of the study were to determine, in the urine and oral samples of young adults, the genotype-specific prevalence of Human Papilloma Virus (HPV) infection, the HPV DNA type-specific prevalence in unvaccinated and vaccinated individuals, and the determinants of HPV infection. Methods: Selected participants were asked to fill in a self-administered questionnaire and to self-collect urine and saliva samples. Results: Among the 1002 participants, 81 (8.1%) resulted positive for HPV DNA. The most common low-risk genotype was HPV 42 (2.2%), followed by HPV 43 (0.8%), and 40 (0.5%). The HPV 51 was the most common high-risk genotype (1.5%) followed by HPV 66 (1%) and HPV 68 (1%), and no participants were infected with HPV genotypes 18, 33, 45. Females, those who have had one or more occasional sexual partner, those who never/rarely/sometimes used condoms during their sexual activity, those with a previous diagnosis of sexually transmitted infection, and those who were not vaccinated were more likely to be tested positive for HPV infection. Conclusions: The low prevalence of genital HPV infections has provided evidence of the effectiveness of HPV vaccination both in vaccinated and not yet vaccinated subjects through herd immunity and indicated its decisive role in the changing epidemiology of circulating HPV genotypes in the population.

## 1. Introduction

Over the last decades, research has been focused on the role of Human Papilloma Virus (HPV) in the development of cervical cancer and other diseases, including cancer of the vulva, vagina, penis, anogenital and oropharyngeal cancers, and genital warts [1,2,3].

The recognition of the role of the HPV in cervical cancer etiology has fostered the achievement of medical advances for its prevention through screening based on HPV testing and vaccination. HPV DNA testing is available for numerous HPV high-risk genotypes and it has been proposed as an alternative to primary screening using cytological testing as it enables early diagnosis of pre-cancerous lesions with higher sensitivity [4]. In addition, immunization is an effective intervention to prevent cervical, vaginal, and vulvar pre-cancerous lesions, penile precancers [5,6], and oral infections [7,8]. To date, three vaccine formulations have been approved in Italy. The bivalent (2vHPV; HPV type 16/18) is indicated in females aged 9–26 years, the quadrivalent (4vHPV; HPV type 6/11/16/18), and the nine-valent (9vHPV; HPV type 6/11/16/18/31/33/45/52/58) were approved also for 9–26-year-old males. The HPV vaccine has been offered free of charge to 12–26 years old females since 2007, and to 12–18 years old males since 2015.

The effects of vaccinations can be monitored by the evaluation of changes in a genotype’s specific HPV prevalence, which may provide information on their impact much earlier than data on the reduction in neoplastic lesions. A rapid evaluation of the vaccination on the circulation of HPV could be used to test the presence of HPV DNA in the urine samples and its detection has been suggested for surveillance and impact studies [9]. Moreover, HPV DNA testing on self-samples has been proposed as an additional strategy to reach non-attendees for screening programs [10].

Despite available data on the effect of vaccination on oral HPV infection and the potential for vaccination programs to change the epidemiology of HPV-related oropharyngeal squamous cell carcinoma, there are limited data regarding oral HPV infections [11,12]. Oral samples collected through several methods, such as saline rinse and gargles or unstimulated whole mouth saliva, have been proposed for oral HPV prevalence studies [13,14], although they do not provide a targeted sample of the biologically relevant cancer site (the oropharynx). Moreover, few studies have been conducted in men and women from the same underlying population and have included sampling at multiple anatomic sites [15,16]. Therefore, the aims of this study were to determine, in urine and oral samples of young males and females in Southern Italy: (1) the genotype-specific prevalence of HPV infection; (2) the HPV DNA type-specific prevalence in unvaccinated and vaccinated males and females; and (3) the determinants of genital and oral HPV infection.

## 2. Materials and Methods

### 2.1. Participants, Sampling, and Data Collection

This study is part of a larger project that was also aimed at investigating the level of knowledge, the attitudes, and the behaviors towards the HPV preventive measures among university students and the associated factors [17]. Briefly, the participants, recruited with a two-stage cluster method between November 2022 and September 2023, were 18–30 years old students attending courses in medicine and surgery, healthcare professions, pharmacy, biology, and sports science in three public universities in Southern Italy.

Sample size calculation was based on expecting a genital HPV prevalence of 20% in those non-vaccinated and of 10% in HPV 16/18 vaccinated, a power of 90%, and an error (two-sided) of 5%. A total sample size of at least 983 individuals was needed and, since the prevalence of oral HPV infection was expected to be 4.5%, the final sample was set at 1500 subjects.

Participants were informed of the objectives of the study, how to return the saliva and urine samples and the written informed consent form to the research team, that the participation was voluntary, that all the information collected would be processed and analyzed anonymously, and that no compensation or gift would be given if they answered the questionnaire or provided the biological samples. Students were asked to fill in a self-administered questionnaire that has already been described [17] and to self-collect a urine and saliva sample. Briefly, the first section was on socio-demographic and general characteristics; the second section investigated level of knowledge about the HPV; the third section was on concerns about the risk of acquiring an HPV infection and related disease or a sexually transmitted disease (STD), and on attitudes about preventive measures (vaccination, Pap test, DNA HPV test) for HPV infection and related diseases; the fourth section asked about the immunization status regarding HPV and the willingness to receive the HPV vaccine; and the last section was on sources of HPV-infection and related preventive measures information, and the need for additional information. Before the questionnaire was distributed, a pilot study was conducted among a small group of 50 students to ensure the questionnaire was easy to understand and to answer.

### 2.2. Urine and Oral Sample Collection

At the time of recruitment study participants were informed that the use of lip cosmetics, drinking, eating, smoking or chewing gums had to be avoided for at least two hours before collection and received an instructions form on how to collect the urine and oral samples with a kit consisting of a urine cup (BD Vacutainer, manufactured by BD Diagnostics, Franklin Lakes, NJ, USA) and a 50 mL tube containing 7 mL PreservCyt Solution (ThinPrep, manufactured by Hologic, Marlborough, MA, USA) for the saliva samples. In particular, the first urine of the day including the first void had to be poured directly into the cup to approximately a 1/3 of its volume and delivered within two hours to the research team. Then, the samples were transferred into a 50 mL tube containing 7 mL of PreservCyt Solution. Oral samples of saliva had to be collected directly into the tube for 1/5 of its volume, ensuring that it was completely resuspended in the fixative. Oral and urine samples were stored at room temperature before processing.

### 2.3. DNA Extraction and HPV Genotyping

Urine and oral samples were placed into a 50 mL tube and centrifuged at 3000× *g* for 10 min, and the supernatant solution was discarded. Cells were resuspended in PreservCyt Solution and transferred into 1.5-mL microcentrifuge tubes.

DNA was extracted from 300 µL of each specimen type using the semi-automated extraction platform Seegene NIMBUS. The HPV genotyping was performed using the Anyplex™ II HPV28 Detection system (Seegene, Seoul, Republic of Korea) that is based on multiplex real-time PCR which allows the simultaneous identification of multiple HPV genotypes using DPO™ (Dual-Priming Oligonucleotides) and TOCE™ (Tagging Oligonucleotide Cleavage and Extension) technologies. The test simultaneously detects, differentiates, and quantizes 28 distinct HPV genotypes, including 19 high-risk (16, 18, 26, 31, 33, 35, 39, 45, 51, 52, 53, 56, 58, 59, 66, 68, 69, 73, 82) and 9 low-risk (6, 11, 40, 42, 43, 44, 54, 61, 70). The amplification of target sequences was performed through PCR multiplex reactions on the CFX96TM Real-time PCR system (Bio-Rad Laboratories GmbH, Munich, Germany). The reaction mix was of a total volume of 20 μL containing 5 μL DNA, 5 μL Mastermix and 5 μL A or B Oligomix. The internal control human β-globin gene was amplified concurrently to ensure the adequate DNA amount and the PCR reaction efficiency. Three positive controls and one negative control provided by the manufacturer were included in each PCR run.

The data recording and interpretation were performed by Seegene viewer Version 3 software according to the manufacturer’s instructions. A positive result (+++/++/+) indicated the presence of HPV DNA, while the negative result (−) indicated the absence. The detection limit of this assay was 50 copies of HPV per reaction. All cases without adequate cellularity were considered invalid results at the HPV DNA test.

### 2.4. Statistical Analysis

Stata Version 18 software was used to perform all statistical analysis. Descriptive analysis was performed to summarize the main characteristics of the sample. Bivariate analysis, using Fisher’s exact test, chi-square, and Student’s *t*-test, investigated the association between several independent variables and HPV infection in urine and oral samples separately. The variables with a *p*-value < 0.25 were included in the final stepwise multivariate logistic regression model to investigate the predictors of HPV infection detected in urine samples. The following independent variables were included in the model: age (continuous), sex (male = 0; female = 1), sexual orientation (heterosexual = 0; other = 1); being smoker (no = 0; yes = 1), consuming alcohol (no = 0; yes = 1), condom use during sexual intercourse (no sexual intercourse-often/always = 0; never/rarely/sometimes = 1), having had sexual intercourse during lifetime (no = 0; yes = 1), current sexual relationship status (no sexual intercourse/none = 0; >one occasional = 1; regular = 2), family history of HPV-related cancers (no = 0; yes = 1), having received at least one HPV vaccination dose (no = 0; yes = 1), prior history of STD (no = 0; yes = 1), and having had oral sex (no = 0; yes = 1). The values of *p* = 0.2 and *p* = 0.4 were used to select variables for inclusion and exclusion in the final model. The results of the stepwise model were measured using Odds Ratios (ORs) and 95% confidence intervals (CIs). A two-tailed *p*-value ≤ 0.05 was considered statistically significant.

## 3. Results

Out of a total of 1490 selected participants, 1002 (67.2%) and 1182 (79.3%) completed the survey and provided a suitable self-collected urine and saliva sample for HPV testing, respectively.

The main characteristics of the participants are shown in Table 1. The mean age was 22.2 years, two-thirds were females, almost all were heterosexual, 21.5% were current smokers, a large majority consumed alcohol. Three-quarters (77.8%) had had a sexual intercourse, 47.3% of them often/always used condoms during their sexual activity, 56.2% currently had a regular partner, 77.2% had had oral sex, 5% had a family history of HPV-related cancers, 1.8% had a prior history of a STD, 48.9% had received at least one HPV vaccination dose, 16.3% and 2.7% of those eligible (≥25 years) had, respectively, undergone a Pap-test or HPV DNA-test. Almost all (94.9%) expressed their willingness to periodically undergo a urine test as an alternative option to clinician- or self-collected cervico-vaginal specimens for an HPV-based cervical cancer screening program.

At the bivariate analysis, the prevalence of urine HPV infection was significantly higher among female (9.8% vs. 4.2%; χ^2^ = 4.11, *p* < 0.01), older participants (mean age of infected = 22.8 years vs. mean age of non-infected = 22.1 years; t = −2.13; *p* = 0.03), who had had a sexual intercourse (9.9% vs. 1.8%; χ^2^ = 15.2, *p* < 0.01), who had one or more occasional partner (19.4% vs. 13.3%; χ^2^ = 21.2, *p* < 0.01), who had never/rarely/sometimes used condoms during their sexual activity (8.5% vs. 6.8%; χ^2^ = 13.2, *p* < 0.01), had had oral sex (10.1% vs. 1.3%; Fisher’s exact *p* < 0.01), had been diagnosed with a STD (33.3% vs. 7.7%; χ^2^ = 15.6, *p* < 0.01), and in current smokers (14% vs. 6.6%; χ^2^ = 11.8, *p* < 0.01) (Table 1). The prevalence was also higher among women who had previously undergone a Pap-test (15.3% vs. 8.5%; χ^2^ = 5.8, *p* = 0.02), whereas no significant difference was found among women who had previously undergone an HPV DNA testing for the early detection of cervical cancer.

The prevalence of urine HPV by sex and HPV vaccination status are presented in Table 2. A total of 81 (8.1%) participants resulted positive for HPV DNA, and 46 of them were infected by a single genotype and 35 by multiple genotypes. Specifically, 29 of them were infected by two genotypes and 6 by three, providing a total of 122 results by 23 genotypes. At least one high-risk HPV genotype was detected in 6.6% participants, whereas 1.5% of them was infected only by low-risk genotypes. Participants infected by one or more of the genotypes included in the 4vHPV and 9vHPV were 0.5% and 1.3%, respectively.

Overall, at the bivariate analysis no significant differences in HPV prevalence were found by the vaccination status. Females showed a higher prevalence compared to males of at least one HPV genotype (9.8% vs. 4.2%), for those at high-risk (8.1% vs. 3.2%), for those at only low-risk (1.7% vs. 0.9%), and multiple HPV infection (4% vs. 2.2%).

The detected urine genotype’s prevalence stratified by sex is displayed in Table 3. Overall, the most common low-risk HPV genotype was the 42 (2.2%), followed by 43 (0.8%), and 40 (0.5%), and no participants were infected with 11. The same trend was observed among females, with 2.5%, 1%, and 0.7% of them infected by 42, 43, and 40, respectively, whereas in males the low-risk HPV genotypes predominantly detected were 42 (1.6%), 54 (0.6%), 6 (0.3%), and 43 (0.3%). HPV 51 was the most common high-risk HPV genotype (1.5%) followed by 66 (1%) and 68 (1%), and no participants were infected with genotypes 18, 33, and 45. HPV 51 was also the most common genotype in both groups of females (1.6%) and males (1.3%).

Table 4 shows the results of the multivariate logistic regression model built to assess which participants’ characteristics influenced the odds of an HPV infection detected in urine. Females (OR = 4.14; 95% CI = 2.03–8.44), those who have had at least one occasional sexual partner (OR = 2.39; 95% CI = 1.16–4.92), those who never/rarely/sometimes used condoms during their sexual activity (OR = 2.14; 95% CI = 1.28–3.55), and those with a previous diagnosis of STD (OR = 3.79; 95% CI = 1.31–10.96), were more likely to be tested positive, whereas those vaccinated against HPV (OR = 0.55; 95% CI = 0.32–0.94) were less likely to be tested positive.

An oral HPV infection was detected in eight participants (0.7%), two males and six females. All six females were also positive for urine HPV infection, and four of them for the same HPV genotype in urine and oral samples. HPV 43 was the only low-risk genotype detected, whereas seven participants were positive for the high-risk HPV genotypes 16, 31, 39, 45, 56, 59, and 66, and six participants had both oral and urine HPV infections. More than half of those who were negative for urine HPV infection (52.8%) and seven out of eight participants tested positive were unvaccinated against HPV, respectively. At the bivariate analysis, the prevalence of oral HPV infection was significantly higher in older subjects (mean age of infected = 24.9 years vs. mean age of non-infected = 22.1 years; t = −3.05; *p* < 0.01), who were current smokers (2.4% vs. 0.4%; Fisher’s exact *p* = 0.01), and who had not been vaccinated against HPV (0.2% vs. 1.5%; Fisher’s exact *p* = 0.04).

## 4. Discussion

The study presents the results of a large project among young adults aged 18–30 years in Southern Italy that has explored the genital and oral circulation of HPV infection and the prevalence of a large set of HPV genotypes in different sites including oral and urine samples.

One of the main findings is that the overall prevalence of HPV infection in urine samples was 8.1%, with a higher prevalence in women (9.9%) compared to men (4.2%). The prevalence in women is similar to that reported in a recent review that showed among healthy women in Europe a value of 9.7% [3], whereas higher prevalence of 19.9% on urine samples has been detected in non-vaccinated women in Norway [18] and of 22.1% in 18–40 years old women in Italy [16]. The few studies on urine in males reported an HPV prevalence of 4.1% in vaccinated and 10% in non-vaccinated 18-year-olds in Finland [19] and of 13.6% in 18–40 years old in Italy [16]. The lower prevalence detected in this study may be the consequence of a more recent picture of HPV circulation, which incorporates the potential effects of vaccination strategies that may have modified the extent of the infection, demonstrating the decisive role of epidemiological surveillance of HPV infections for monitoring and evaluation of the real-world effectiveness of the available vaccines and immunization strategies. Indeed, a declining trend has been observed in HPV infections in 20–25 years old German women 10 years after the introduction of the vaccination [20] and in US women in several age groups for vaccine-type HPV infections from 2003 to 2018 [21]. Moreover, in the Campania and Calabria regions, the coverage for at least one dose ranged, respectively, from 41.7% and 49.8% for girls who were 12 years old to 67.5% and 76.1% for those aged 16 years in the year 2021, with lower coverages in the same male cohorts (5.1–23.5% and 50.6–71.1%) [22]. The self-reported HPV vaccination status in this study only partially confirmed these data and the lower incidence of HPV positivity among unvaccinated participants could be due to a possible strong role of herd immunity.

The pattern of HPV genotypes circulation has provided an impressive scenario that is worthy of thoughtful appraisal. It is noticeable that most of the burden of infection is related to high-risk genotypes which account for 60% of all detections and is observed in more than 80% of those infected. Moreover, the prevalence of multiple infections by two or three genotypes is substantial and concerning, given that they are more frequently reported in cervical lesions [23]. However, probably the most interesting result is the distribution of the different investigated genotypes, which showed the very low prevalence of those included in the 2vHPV and 4vHPV, with only five infections (0.5%) and no detection of the genotypes 11 and 18. The decline of the circulation of the genotypes included in the first two vaccines confirms previous studies performed after the implementation of HPV vaccination strategies, in which the most frequent high-risk genotypes (HPV 16–18) have shown substantial frequency decrease [23,24,25]. It is also remarkable that the other high-risk genotypes included in the 9vHPV were not very spread, whereas the highest prevalence among high-risk genotypes were found for HPV 51, 66, and 68. This low prevalence is reassuring, since infection by these genotypes has been more frequently associated with cervical lesions in women undergoing cervical cancer screening in Portugal [26], and it cannot be excluded that, maintaining a cautious approach, this decline is at least partly a consequence of the gender neutral 9vHPV vaccination strategy. In all analyses, HPV genotypes were less frequent in vaccinated subjects, although not always significantly. A possible explanation may be the low power of the test, considering that the prevalence was low for all genotypes, even when they were aggregated according to several criteria. However, it cannot be excluded, as reported in other studies [20,27,28,29,30], that the low prevalence also among those unvaccinated is a consequence of the herd protection effect, attributable to the reduced circulation of genotypes included in the vaccines. As regards to the higher prevalence of high-risk non-vaccine HPV genotypes, since no pre-vaccination data are available on the circulation of these genotypes in this area, we do not know if the prevalence has been constant or has increased because of type replacement resulting from the reduced competition with HPV genotypes included in the vaccines [31]. Confirmation of these genotype’s prevalence pattern in other studies would have valuable implications for the development of future HPV vaccines.

The results should be interpreted also considering that HPV circulation has been investigated in urine samples. There is consensus that the advantage of a noninvasive self-sampling procedure is coupled with, especially in women, strong potential for application in screening programs and epidemiological investigations. Indeed, the World Health Organization recommended self-sampling methods as a complementary option to cervical screening that could address gaps in current coverage and aid in reaching the target of 70% coverage by 2030 [32], and a recent meta-analysis [33] showed that self-sampling procedures had the potential to increase participation in cervical cancer screening programs among general and under-screening populations. Indeed, several studies have detected similar sensitivity and specificity for cervical intraepithelial neoplasia grade 2 or higher (CIN2+) in self-collected first-void urine samples versus clinician- or self-collected cervical samples [34,35,36,37,38,39,40,41,42]. Moreover, it has proved to be a highly practical means to ascertain the population-level impact of HPV vaccination in young women [43]. This is confirmed by the findings of this study, since almost all participating women would be willing to undergo urine tests as an alternative to traditional methods for cervical cancer screening programs and have provided valuable results for the assessment of HPV circulation in young healthy subjects. In contrast to women, monitoring HPV prevalence in males by urine specimens do not seem to be optimal for the detection of anogenital HPV infections due to a low human genomic DNA content compared to other urogenital sites [44,45], and the results of this study showed lower HPV prevalence in males’ urines. Therefore, interpretation of results in males should be cautious and comparisons with studies which used the same specimens would be appropriate. However, it remains a relevant option for sampling the general population in the youngest age groups for epidemiological purposes.

One of the aims of the study was to ascertain predictors of HPV infection detected by urine. As expected, sexual risky behaviors, such as a higher number of occasional sexual partners or no regular use of a condom, as well as a previous diagnosis of STD, were significant predictors of HPV infections. Moreover, the finding that HPV infections were significantly more likely in females should be interpreted with caution, since, as previously addressed, it may have been influenced by the detection methods, which were less appropriate for the males. One of the most interesting results of the multivariate analysis pertains to the significantly lower risk of HPV infection detected in vaccinated subjects, which provides real world evidence of the effectiveness of the vaccination strategies, as well as the relevant role of repeated HPV prevalence investigations for monitoring the effect of vaccination on the epidemiology of the infection.

An oral HPV prevalence of 0.7% has been observed. A substantial variability has been reported ranging from 0.3% in Italy in healthy adults [46] to more than 10% [3,45,47,48], with an overall value of 7.7% reported by a meta-analysis conducted in 18 years and older healthy males and females [12]. This variability has been attributed to differences in the study populations, geographic variations, methods of collection of specimen and HPV detection, and in the investigated HPV genotypes. It is worth underscoring, however, that studies that have investigated trends of oral HPV prevalence over time have demonstrated a steady decrease in vaccinated [7] and non-vaccinated subjects [44], specifically driven by a reduction in HPV vaccine genotypes. Therefore, the low prevalence found in this study might indirectly confirm the role of vaccination also on HPV infection prevalence in the oral cavity, which is confirmed by the observed significantly lower risk of oral HPV infection among vaccinated subjects.

It is worthy of regard that six out of eight oral HPV infections were found in subjects who were positive also at urine samples, and that in four out of these six cases the same genotype was found in the two samples. There are sparce data on concurrent oral and genital HPV infections [49,50] and recently this association was found more in men who have sex with men, compared to heterosexual males [51]. Studies among healthy women have suggested that HPV oral and genital dual infections are not independent, although HPV genotype specific concordance has been found to be low [52,53,54]. However, the importance of several demographic, specifically younger age and low socioeconomic status, and behavioral factors, specifically oral sex practices, has been observed in the development of dual and concordant genotype HPV infection in women [53]. Therefore, although due the small number of oral HPV infections found in this study no definite conclusions can be drawn, the high genotype concordance observed (66.7%) in dual infections is worth of further investigation.

The findings of this study should be interpreted by addressing some potential limitations. First, the cross-sectional design does not allow us to distinguish persistent genital HPV infections, a more reliable indicator of the risk of HPV related diseases, and the extent of HPV prevalence should be interpreted as an indication of the circulation of this infection after the introduction of the vaccination. Also, the design precludes causal inferences on the determinants of HPV infection. Second, careful consideration of the self-reported nature of the data should be given particularly to the information on vaccination and sexual behavior, which could have been influenced by desirability bias, although the anonymity of the questionnaire might have minimized this issue. Third, regarding HPV testing, given the described limits of urine test on males, the results should be interpreted only in comparison with analogous studies using the same methodology, whereas the low prevalence of HPV infection detected in oral samples has precluded a detailed analysis of the distribution and determinants of oral infections. Despite these limitations, the results have produced valuable new knowledge with relevant public health implications.

## 5. Conclusions

The low prevalence of genital HPV infections has provided evidence of the effectiveness of the HPV vaccination strategies both in vaccinated and not yet vaccinated subjects through herd immunity and indicated its decisive role in the changing epidemiology of circulating HPV genotypes in the population. Moreover, this study showed that oral HPV infections were not widely spread in the young adults and research is needed to have sharper insight on the role of dual and concordant genital and oral HPV infections. Data provided by repeated epidemiologic studies investigating HPV genotypes dynamics would be invaluable in the assessment of vaccination effectiveness over time and in orienting research on HPV genotypes to be included in future vaccines.

## Figures and Tables

**Table 1:** Main characteristics of the participants and the associated positivity for urine HPV infection.

		Total (*n* = 1002)		Tested Positive (*n* = 81; 8.1%)		
Characteristics	Option	N	%	N	%	
Age		22.2 ± 2.7 (18–30) *		22.8 ± 2.6 (18–30) ^a^ 22.1 ± 2.6 (18–30) ^b^		t = −2.13; *p* = 0.03
Gender	Female	690	65.4	68	9.8	χ^2^ = 4.11; *p* < 0.01
	Male	312	34.6	13	4.2	
Sexual orientation	Heterosexual	920	92.8	75	8.1	χ^2^ = 0.07; *p* = 0.06
	Asexual/bisexual/gay/lesbian/pansexual	82	7.2	6	7.3	
Being smokers °	No	755	78.5	50	6.6	χ^2^ = 11.8; *p* < 0.01
	Yes	207	21.5	29	14	
Consuming alcohol	No	111	11.1	4	3.6	χ^2^ = 3.37; *p* = 0.07
	Yes	891	88.9	77	8.6	
Having had sexual intercourse during lifetime °	No	222	22.2	4	1.8	Fisher’s exact *p* < 0.01
	Yes	779	77.8	77	9.9	
Current sexual relationship status °	Regular partner	544	56.2	51	9.4	χ^2^ = 21.2; *p* < 0.01
	≥one occasional partner	67	6.9	13	19.4	
	No sexual intercourse/none	358	36.9	14	3.9	
Condom use during sexual intercourse ^	Never/rarely/sometimes	305	52.7	45	8.5	χ^2^ = 13.2; *p* < 0.01
	Often/always	474	47.3	32	6.8	
Having had oral sex during lifetime	No	228	22.8	3	1.3	Fisher’s exact *p* < 0.01
	Yes	774	77.2	78	10.1	
Family history of HPV-related cancers	No	952	95	74	7.8	χ^2^ = 2.48; *p* = 0.11
	Yes	50	5	7	14	
Having received at least one HPV vaccination dose	No/do not remember	512	51.1	42	8.2	χ^2^ = 0.02; *p* = 0.89
	Yes	490	48.9	39	7.9	
Reporting a prior history of a sexually transmitted Infection °	No	978	98.2	75	7.7	χ^2^ = 15.6; *p* < 0.01
	Yes	18	1.8	6	33.3	

° Number for each item may not add up to total number of study population due to missing value. * Mean ± Standard deviation (range). ^a^ Mean ± Standard deviation (range) of infected participants. ^b^ Mean ± Standard deviation (range) of non-infected participants. ^ Among those who had had a sexual intercourse.

**Table 2:** Prevalence of urine HPV by sex and HPV vaccination status in the study population.

HPV Genotypes	Total N = 1002		Female N = 690		Male N = 312		Unvaccinated Participants N = 512		Vaccinated Participants N = 490	
	N	%	N	%	N	%	N	%	N	%
Negative subjects	921	91.9	622	90.2	299	95.8	470	91.8	451	92.1
Any HPV	81	8.1	68	9.8	13	4.2	42	8.2	39	7.9
							χ^2^ = 0.02; *p* = 0.89			
At least one high-risk	66	6.6	56	8.1	10	3.2	37	7.2	29	5.9
							χ^2^ = 0.336; *p* = 0.56			
Only low-risk	15	1.5	12	1.7	3	0.9	10	1.9	5	1
							Fisher’s exact *p* = 0.22			
2vHPV	2	0.2	2	0.3	0	0	1	0.2	1	0.2
							Fisher’s exact *p* = 0.67			
4vHPV	5	0.5	5	0.7	0	0	3	0.6	2	0.4
							Fisher’s exact *p* = 0.52			
9vHPV	13	1.3	13	1.9	0	0	9	1.7	4	0.8
							Fisher’s exact *p* = 0.19			
Multiple	35	3.5	28	4	7	2.2	22	4.3	13	2.6
							χ^2^ = 2.01; *p* = 0.16			

**Table 3:** Urine HPV genotypes distribution by sex in the study population.

	Tested Positive N = 81		Total N = 1002		Female N = 690		Male N = 312	
Low-risk	N	%	N	%	N	%	N	%
6 ^b,c^	3	3.7	3	0.3	2	0.3	1	0.3
11 ^b,c^	0	0	0	0	0	0	0	0
40	5	6.2	5	0.5	5	0.7	0	0
42	22	27.2	22	2.2	17	2.5	5	1.6
43	8	9.9	8	0.8	7	1	1	0.3
44	3	3.7	3	0.3	3	0.4	0	0
54	4	4.9	4	0.4	2	0.3	2	0.6
61	1	1.2	1	0.1	1	0.1	0	0
70	2	2.4	2	0.2	2	0.3	0	0
**High-risk**								
16 ^a,b,c^	2	2.4	2	0.2	2	0.3	0	0
18 ^a,b,c^	0	0	0	0	0	0	0	0
31 ^c^	2	2.4	2	0.2	2	0.3	0	0
33 ^c^	0	0	0	0	0	0	0	0
35	0	0	0	0	0	0	0	0
39	9	11.1	9	0.9	8	1.1	1	0.3
45 ^c^	0	0	0	0	0	0	0	0
51	15	18.5	15	1.5	11	1.6	4	1.3
52 ^c^	3	3.7	3	0.3	3	0.4	0	0
56	6	7.4	6	0.6	3	0.4	3	1
58 ^c^	2	2.4	2	0.2	2	0.3	0	0
59	4	4.9	4	0.4	4	0.6	0	0
66	10	12.3	10	1	9	1.3	1	0.3
68	10	12.3	10	1	8	1.1	2	0.6
69	1	1.2	1	0.1	1	0.1	0	0
73	6	7.4	6	0.6	5	0.7	1	0.3
82	4	4.9	4	0.4	3	0.4	1	0.3

^a^ Included in the 2vHPV. ^b^ Included in the 4vHPV. ^c^ Included in the 9vHPV.

**Table 4:** Multivariate logistic regression analysis for factors associated with positive test for urine HPV infection.

Variable	OR	SE	95% CI	*p*
Model. HPV Infection detected in Urine Samples				
Sex				
Male	1 *			
Females	4.14	1.5	2.03–8.44	<0.01
Condom use during sexual intercourse				
No sexual intercourse-Often/always	1 *			
Never/rarely/sometimes	2.14	0.55	1.28–3.55	<0.01
Reporting a prior history of a sexually transmitted infection				
No	1 *			
Yes	3.79	2.05	1.31–10.96	0.01
Current sexual relationship status				
No sexual intercourse/none	1 *			
≥one occasional partner	2.39	0.88	1.16–4.92	0.02
Regular partner	Backward elimination			
Having received at least one HPV vaccination dose				
No	1 *			
Yes	0.55	0.15	0.32–0.94	0.03
Consuming alcohol				
No	1 *			
Yes	2.48	1.7	0.65–9.51	0.14
Having had sexual intercourse during lifetime				
No	1 *			
Yes	5.16	5.9	0.55–47.9	0.15
Having had oral sex				
No	1 *			
Yes	2.48	1.7	0.65–9.51	0.18
Age (continuous)	1.05	0.05	0.96–1.15	0.25
Being smoker				
No	1 *			
Yes	1.28	0.35	0.75–2.19	0.35

* Reference category.

## Data Availability

The data presented in this study are available upon request from the corresponding author.
